# Insects as an alternative protein source for poultry nutrition: a review

**DOI:** 10.3389/fvets.2023.1200031

**Published:** 2023-08-17

**Authors:** Imen Belhadj Slimen, Houari Yerou, Manel Ben Larbi, Naceur M’Hamdi, Taha Najar

**Affiliations:** ^1^Laboratory of Materials Molecules and Applications, Preparatory Institute for Scientific and Technical Studies, Tunis, Tunisia; ^2^Department of Animal Sciences, National Agronomic Institute of Tunisia, Carthage University, Tunis, Tunisia; ^3^Department of Agronomic Sciences, SNV Institute, Mustapha Stambouli University, Mascara, Algeria; ^4^Laboratory of Geo Environment and Development of Spaces, Mascara University, Mascara, Algeria; ^5^Higher School of Agriculture, University of Carthage, Mateur, Tunisia; ^6^Research Laboratory of Ecosystems and Aquatic Resources, National Agronomic Institute of Tunisia, Carthage University, Tunis, Tunisia

**Keywords:** insect meal, broiler chickens, laying hens, feed, digestibility, safety, immune system, bioactive molecules

## Abstract

This review summarizes the most relevant scientific literature related to the use of insects as alternative protein sources in poultry diets. The black soldier fly, the housefly, the beetle, mealworms, silkworms, earthworms, crickets, and grasshoppers are in the spotlight because they have been identified as an important future source of sustainable animal proteins for poultry feeding. Insect meals meet poultry requirements in terms of nutritional value, essential amino acid composition, nutrient digestibility, and feed acceptance. Furthermore, they are enriched with antimicrobial peptides and bioactive molecules that can improve global health. Results from poultry studies suggest equivalent or enhanced growth performances and quality of end-products as compared to fish meal and soybean meal. To outline this body of knowledge, this article states established threads of research about the nutrient profiles and the digestibility of insect meals, their subsequent effects on the growth and laying performances of poultry as well as the quality of meat, carcass, and eggs. To fully exploit insect-derived products, the effects of insect bioactive molecules (antimicrobial peptides, fatty acids, and polysaccharides) were addressed. Furthermore, as edible insects are likely to take a meaningful position in the feed and food chain, the safety of their derived products needs to be ensured. Some insights into the current knowledge on the prevalence of pathogens and contaminants in edible insects were highlighted. Finally, the effect of insect farming and processing treatment on the nutritive value of insect larvae was discussed. Our overview reveals that using insects can potentially solve problems related to reliance on other food sources, without altering the growth performances and the quality of meat and eggs.

## 1. Introduction

“Feed-food competition” was defined as “the tensions and trade-offs between two alternative uses for edible crops: direct consumption by humans versus feeding livestock” (1). However, feed-food competition includes the use of production resources, such as land, wild fish, and water, and labor, capital, and ecosystem services. The allocation of these resources between all their possible uses is often determined by which end use is most profitable. Mottet et al. (2) estimated that 0.4 billion ha of cropland is oriented to feed production, in a competing way with human food. In the same context, Alexander et al. (3) estimated that feed crops and processed feed (i.e., oilseed cakes) account for 27% of livestock feed and 30% of crop consumption. Mottet et al. (2) reported similar estimations. If human food accounts for 45% of crops, the quantity of crops available for direct human consumption could rise by two-thirds if feed crops and processed feed were no longer given to livestock (1).

**Figure 1:**
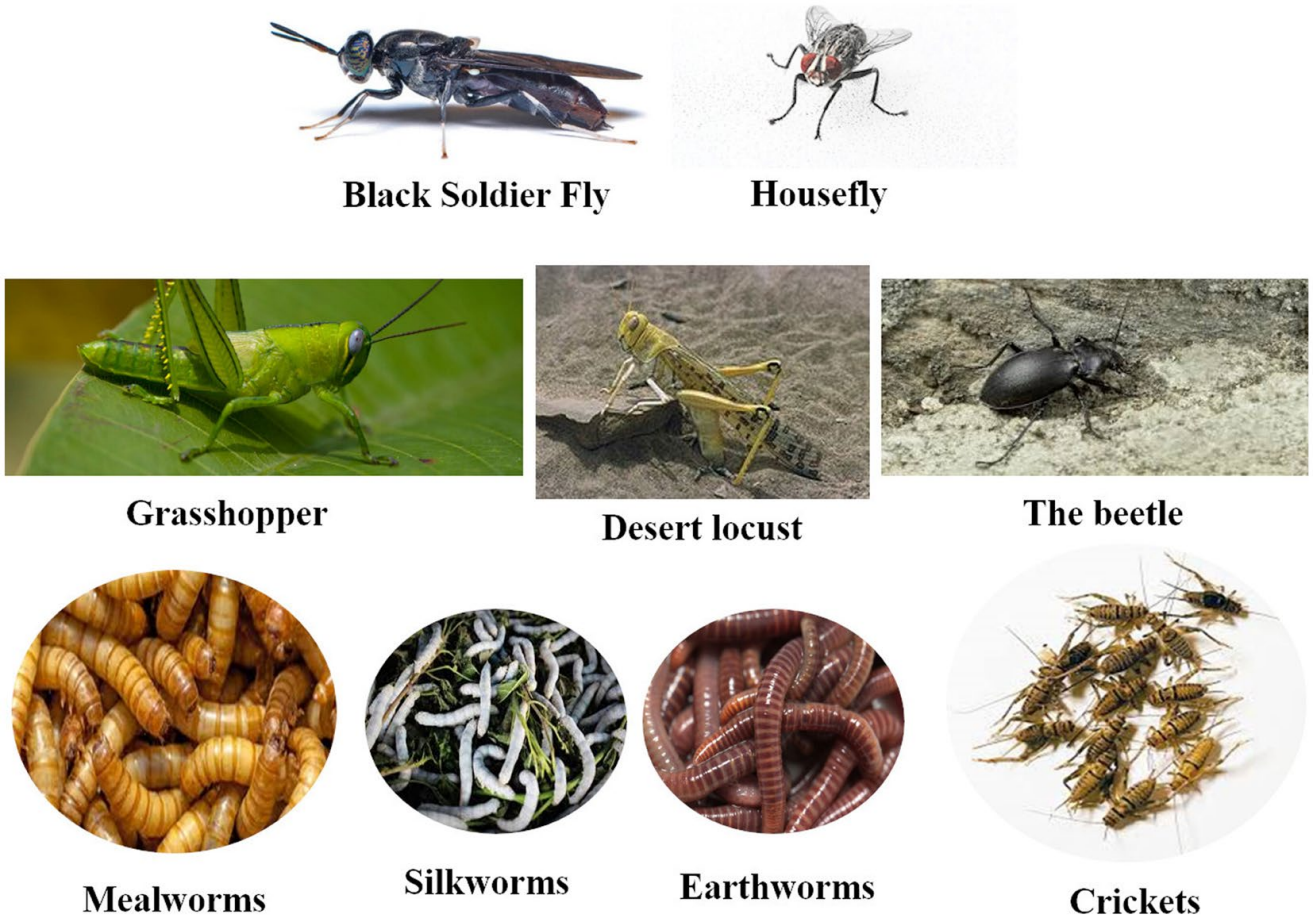
Some insects used in poultry feeding.

Digging in and scratching the ground is a natural behavior of poultry. This innate instinct is triggered by the numerous ‘delicacies’ that are found underground, including worms and insect larvae. Birds prefer insect-based diets, probably because of their taste and nutritive value. In this trend, insects can provide an alternative protein source for poultry production. They can generate income for both industrial and smallholder farmers and are potentially able to reduce feed-food competition. Recently, there has been an escalating interest in insects as an alternative protein source in poultry diets. There are 13 types of insects that have been identified and confirmed to be incorporated in poultry feeding. Among them, eight insects were approved by the EU regulations to be used as food (4) and feed (text of the commission regulation (EU) 2017/893 of 24 Mai 2017 amending annexes I and IV to Regulation (EC) No 999/2001 of the European Parliament and of the council, as well as the annexes X, XIV and XV to the commission regulation (EU) No 142/2011 as regards the provisions on processed animal protein). The most studied insects (Figure 1) include mainly the black soldier fly (BSF), and other insect types such as the housefly (HF), the mealworm (MW), the beetle, the locusts, the silkworm (SW), and the cockroach (5–7).

Insect meal is a low-cost and environmental-friendly ingredient. It contributes to decreasing environmental pollution as organic wastes are used in insect breeding (8–10). Insect meals are considered as potential substitution candidates for soybean meal (SBM) and fish meal (FM) since they have been used as partial or total substituents (9–13) without adverse effects on production performances or product quality.

This review article provides an overview of the nutritional value and digestibility of some insect meals, as well as their effects on animal growth performance, meat and egg quality, and overall health. Concerns about the safety of rearing insects or feeding insect-based diets are also addressed.

## 2. Nutritive value

Increasing meat production requires the expansion of agricultural areas and the increase of feed and water consumption. The reduction of arable lands, global climate change, and the shortage of freshwater resources make it hard to meet the escalating demand for proteins. In this context, it makes sense to look for alternative and sustainable protein sources, which may contribute to global food security. Edible insects indeed require much less land than conventional livestock (14), but they also produce much fewer greenhouse gases (14) allowing them to mitigate the negative effects of climate change (15). Indeed, insects have a high food conversion ratio (FCR). To produce the same amount of proteins, crickets, for example, require half as much feed as broiler chickens, four times less feed than sheep, and twelve times less feed than cattle (16). Moreover, insect meals are a good source of protein, energy, digestible essential and nonessential amino acids, and saturated (SFA), monounsaturated (MUFA), and polyunsaturated fatty acids (PUFA), in addition to vitamins and minerals.

The black soldier fly (BSF) is a common and widespread fly of the family *Stratiomyidae*. BSF meal is a naturally nutritious, protein-packed, and calcium-rich source. The concentration of crude protein (CP) in BSF meal varies from 35 to 61%, and the content of crude fat (*CF*) ranges from 7 to 42% (Table 1). High concentrations of lauric and palmitic acids are also reported (48). Methionine, lysine, threonine, and valine contents were found to be between 0.08 and 0.90% (21, 22, 49), 0.34 and 3.30% (19–21), 0.22 and 2.26% (21, 22), and 0.33 and 3.38% (21, 22) respectively. The concentration of calcium ranges from 1.21% (22) to 4.39% (17), and that of phosphorous varies from 0.74% (23) to 0.95% (22). The nutritive value of BSF meal depends on the rearing substrate, the age of harvesting, and the technology of processing. The pressing and defatting treatments could decrease fat content, from 37% (original) to 27.36% (steamed), 17.05% (methanol extracted), 17.95% (normal pressing), or 13.05% with heated pressing (50).

**Table 1:** Chemical composition of some common insect meals used in poultry nutrition (DM basis).

Common name	Scientific name	Chemical composition				References
		CP (%)	EE (%)	Ca (%)	*P* (%)	
Black soldier fly	*Hermetia illucens*	33–60.8	6.84–42.27	1.21–4.39	0.74–0.95	17–23
House fly	*Musca domestica*	40.12–63.99	2.7–27.9	0.49	1.09	24–28
Cricket/grasshopper/locust	*Acheta/Orthoptera/Schistocerca*	47.73–65.4	2.58–27.1	0.02	0.37	29–33
Silkworm	*Bombyx mori*	45.87–71.9	2.5–30.3	0.1–0.2	0.7–1.1	34–38
Mealworm	*Tenebrio molitor*	27.15–53	3.6–38.3	0.04	0.70	34, 39–41
Earthworm	*Eisenia fetida Lumbricus terresstris*	41.42–65.68	2.25–18.5	0.04–6.3	0.15–2.75	42–46
Cotton leaf worm	*Spodoptera littoralis*	51.2	30.1	-	-	47

DM, dry matter; CP, crude protein; EE, ether extract; Ca, calcium; P, phosphorous.

The housefly (HF) can be found in all countries and can be reared on animal manure and food waste. HF meal contains considerable amounts of CP and *CF* ranging from 40 to 64%, and from 2.5 to 28%, respectively (Table 1). In HF larvae, the content of CP decreases with age, whereas the content of *CF* increases (51, 52). Lysine and methionine are the most limiting amino acids in poultry nutrition. Both of these amino acids are available in high amounts in HF meal (53). Interestingly, the amino acid profile of HF meal resembles that of FM. Similarly to BSF meal, the processing method of the insect impacts the nutritive value of the meal (52).

Mealworms (MW) can be found worldwide where warm, dark, and damp places are available. MW meal is considered a good source of protein and fat, with a CP amount ranging from 27 to 54%, and *CF* content from 4 to 34% (Table 1). Values of ash, crude fibers, and energy in MW larvae range from 3.0 to 4.5% (53, 54), 5.0 to 8.8% (53, 54), and 1,378 to 4029.63 Kcal/Kg DM (45, 49), respectively. As compared to SBM, MW larvae had higher CP, *CF*, and ADF (11). Methionine + cysteine, lysine, leucine, isoleucine, histidine, threonine, valine, and arginine contents are higher in SBM, whereas MWs had greater tryptophan content (11). Moreover, MW larvae contain low amounts of Ca (434.59 mg/Kg) and high levels of K and P [9479.73 and 7060.70 mg/Kg, respectively (41)]. Consequently, the Ca/P ratio is not suitable for poultry production, especially for hens, and Ca supplementation is recommended (5). In addition to these minerals, Zn, Fe, and Cu are found in MW in considerable amounts [104.28, 66.87, and 13.27 mg/kg, respectively (41)].

Crickets and grasshoppers are also valuable protein sources for poultry. The amount of CP ranges from 48 to 65% and *CF* is between 3 and 21% (Table 1). Of course, these contents varied between African grasshoppers (*Acanthacris ruficornis*), Chinese grasshoppers (*Acrida cinerea*), wild edible grasshoppers (*Ruspolia nitidual*), short-horned grasshoppers (*Oxya hyla hyla*), and desert locusts (*Schistocerca gregaria*) (32). The crude fiber content varies between 1.06 and 9.21% (55, 56). Ojewola and his coauthors reported GE values of 1,618 and 1917 Kcal/Kg (55, 57), and Sun and his collaborators reported ME values ranging from 3,923 to 4,018 Kcal/Kg (58). The amino acid (AA) content matched well with those of FM. Regarding methionine, cysteine, and lysine, the relative contents were evaluated at 1.70, 0.69, and 3.79% on DM basis, respectively (30).

.In addition to crickets, earthworms (EW) are a good source of protein, energy, and amino acids (42, 43, 49). The CP content of EW meal ranges from 41 to 66%, and *CF* is between 3.5 and 18% (Table 1). Important contents of lysine and methionine were recorded in EWs (45, 60). However, it is important to mention that these values depend on the freshness and dryness of the EWs.

The nutritional value of a feed ingredient is determined by its chemical composition and the bioavailability of its nutrients. These two factors impact the use of nutrients in forming cells and tissues and in performing the maintenance and production functions of the bird body. Hence, it is important to examine the content of balanced proteins; the profile of amino acids, especially the essential ones; the content and composition of fats; and the amounts of vitamins and minerals. Nowadays, there is evidence that insects are rich in nutrients (proteins, energy, fat, and ash), including those considered essentials for the growth and production of birds. Scientific data indicate that edible insects are a source of complete animal protein and contain all essential amino acids. They can therefore be utilized in poultry diets. Also, they can be incorporated in cereal-based poultry feed, since cereals are poor in essential amino acids such as lysine, threonine, and tryptophan. In addition, insects are good sources of lysine, which is the reference amino acid in poultry and all remaining essential amino acids are related to it.

Adult insects have lower ME content than larvae and pupae because of the decrease of the crude fat content due to age. The nutritional value of lipids is related to the content and profile of their fatty acids. Among saturated fatty acids, insects have high contents of palmitic acid (C16:0), which is involved in stimulating blood cholesterol, especially the LDL fraction. Insects contain also both MUFA and PUFA, especially oleic (C18:1) and linoleic (C18:2) which were noticed for their positive effects on broiler meat quality (61, 62). Knowledge of nutrients and the amounts in which they occur in various insect species indicates the possibility to switch to insect-based diets in poultry feeding. However, knowledge of the best combination ratios of insect proteins/cereals proteins should be deepened.

## 3. Digestibility

Some decades ago, BSF larvae were reared on beef cattle feces and urine. They were then collected, dried, and fed to growing pigs. In an assay, researchers demonstrated that BSF larvae are a suitable ingredient for pig diets, although the fat content may affect both the palatability and digestibility of the diet (63). Many other reports have confirmed that using insect meals as an alternative protein source in broilers resulted in a reduced FCR and improved meat quality indicators. These findings may be attributed to the enhanced digestibility of dry matter, crude protein, crude fiber, ether extract, and ash (34, 64). Cullere et al. (65) reported no significant differences in DM, OM, and starch digestibility between growing broilers that received BSF meal and controls. However, they calculated superior EE digestibility for the group of broilers that received 15% defatted BSF larvae meal. The total tract digestibility (TTD) of DM and ash did not differ significantly between housefly larvae and pupae meals. However, AME and EE were different (66). Bosch et al. (67) evaluated the *in-vitro* OM digestibility of MW larvae to be 91.5%. De Marco et al. (49) compared MW and BSF meals regarding the TTD of nutrients and energy, and concluded that there were no significant differences between the two insect meals for all nutrients, except for the value of EE which was higher in MW larvae meal than in BSF meal (Table 2). Similarly, no statistical difference was reported between these two insect meals for AME (4026.94 and 4151.14 kcal/kg DM for MW and BSF, respectively) and AMEn (AMEn = 3826.31 and 3964.84 kcal/kg DM for MW and BSF, respectively).

**Table 2:** Total tract apparent digestibility of some insect meals (%).

Substrate	Digestibility (%)						References
	OM	DM	N	CP	EE	GE	
Insect meal							
BSF Larvae	84.3	53	89.7	51	88	69	49, 67
Mealworm	66–91.5	60	91.3	60	99	64	49, 67
HF Larvae	-	81	-	69–98	94	77	69, 70
HF Pupae	-	83	-	79	98	-	70
House cricket	88	-	91.7	-	-	-	67
Reference							
SBM	80.6	-	94.7	98	-	-	64, 67

OM, organic matter; DM, dry matter; N, nitrogen; CP, crude protein; EE, ether extract; GE, Goss energy; BSF, black soldier fly; HF, housefly; SBM, soybean meal.

The coefficient of total tract digestibility (CTTD) of crude fibers (*CF*) of the housefly meal (*Musca domestica*) ranges from 0.58 to 0.62 (66). The CTTD of neutral detergent fibers (NDF) was evaluated at 0.87 and that of acid detergent fibers (ADF) varied from 0.35 to 0.67 for larvae and pupae meals (66). In poultry, dietary fibers are associated with reduced digestion as they lower the transit rate of almost all nutrients in the upper digestive tract, and thus a longer time for digestion was registered in the lower digestive tract (68). Bosch et al. (67) reported an *in vitro* nitrogen digestibility of MW larvae of 91.3%. The proteins of the housefly larvae meal (*Musca domestica*) are highly digestible in broilers. Zuidhof et al. (69) and Hwangbo et al. (64) evaluated the TTDs for CP of housefly larvae meal at 98.8 and 98%, respectively. Lower values were obtained by Pieterse and Pretorius (66) who compared between pupae and larvae meals of houseflies, and calculated TTDs of 79 and 69%, respectively. The TTD values reported in the literature of the CP of pupae meal are comparable or higher to those of soybean meal (Table 2). Similarly, Cullere et al. (65) tested the digestibility of CP from BSF in growing broiler quail. They concluded that there are no significant differences between the groups that received 10 and 15% defatted BSF larvae meal compared to the control group.

The AA of insects are very digestible in broilers. The CTTD of both essential and non-essential amino acids ranges from 0.83 to 1.00 (66). The reported TTD values of essential amino acids of houseflies are higher than those of soya oil cake meal. De Marco et al. (49) stated that the average of the apparent ileal digestibility coefficient (AIDC) of the AAs was greater in MW (0.86) as compared to BSF (0.68). Zuidhof et al. (69), Hwangbo et al. (64), and Pieterse and Pretorius (66) concluded that the CTTDs for certain essential amino acids (threonine, lysine, methionine, isoleucine, phenylalanine, and leucine) and all non-essential amino acids, except tyrosine, calculated for pupae meal are significantly (*p* < 0.05) higher than those calculated for larvae meal. In another study, Hall et al. (53) compared the AIDC and the true ileal digestibility coefficient (TIDC) of AA from housefly larvae and those from fish meal. They concluded that these two protein sources had similar values of AIDC and TIDC, and these values are close to those reported in the literature (71, 72). Nevertheless, in another study, Ravindran et al. (73) reported intermediate digestibility coefficients of amino acids in FM. This notable mismatch could be attributed to the method used to assess digestibility, or to the drying process which may damage the AAs. Grasshoppers were also reported for the elevated digestibility of their AAs. The true amino acid digestibility (TAAD) coefficients of methionine, cysteine, and lysine in grasshoppers were 0.97, 0.84, and 0.95, respectively, determined in cecectomized roosters (30). According to this latter study, the average TAAD coefficients of field crickets (92.9%) were higher than that of fish meal (91.3%), and the TAAD coefficients for EAAs in field crickets ranged from 82% for cysteine to 99% for asparagines.

It is worth stating that the findings regarding TTDs of CP and amino acids of larvae and pupae meals are surprising since pupae hold a chitin layer, an indigestible polysaccharide found in the cuticle of insects, which may decrease the availability of nutrients once fed to broilers (74). Decreased CTTD for CP from the housefly larvae meal may be due to overheating during processing, which is the factor most often responsible for damaging the quality of proteins and reducing the digestibility of amino acids (75). Indeed, the milling process of the pupae may be responsible for reducing the particle size. Thus, the surface area available for digestion is increased and the substances of the chitin layer are more available to the animal (76). It is certain that the drying and processing of insects’ larvae affect the chemical composition and the digestibility of nutrients. Raw MWs had a higher digestibility of CP compared to cooked ones (77). However, it is worth stating that using raw insects in poultry diets maybe not be feasible because of storage, transportation, and product safety issues. Oven-dried larvae at 60°C had a greater AA digestibility than microwave-dried ones (78). Despite the cooking process, the quality of insect proteins depends on the stage of insect production (77). In fact, the amount of chitin is higher in adult crickets than in larvae. Consequently, the digestibility of adult crickets is reduced (77). In the same trend, an assay was carried out to evaluate the effect of including 0.05% cricket chitosan or 0.05% cricket chitin in Cobb 500 male broiler chickens. The results showed that the intestinal morphology was negatively affected and the expression of some nutrient transporters (PepT1, EAAT3, SGLT1, and SGLT5) was downregulated (79).

The high content of digestible CP and amino acids in insect-derived products introduces them as a potent future-proof solution to upgrade protein self-sufficiency in animal feed. Since they are enriched in essential and limiting amino acids (lysine, threonine, methionine, and tryptophan), insects meet the nutritional requirements of poultry and contribute to the development of a highly formulated feed.

## 4. Bioactive substances

**Figure 2:**
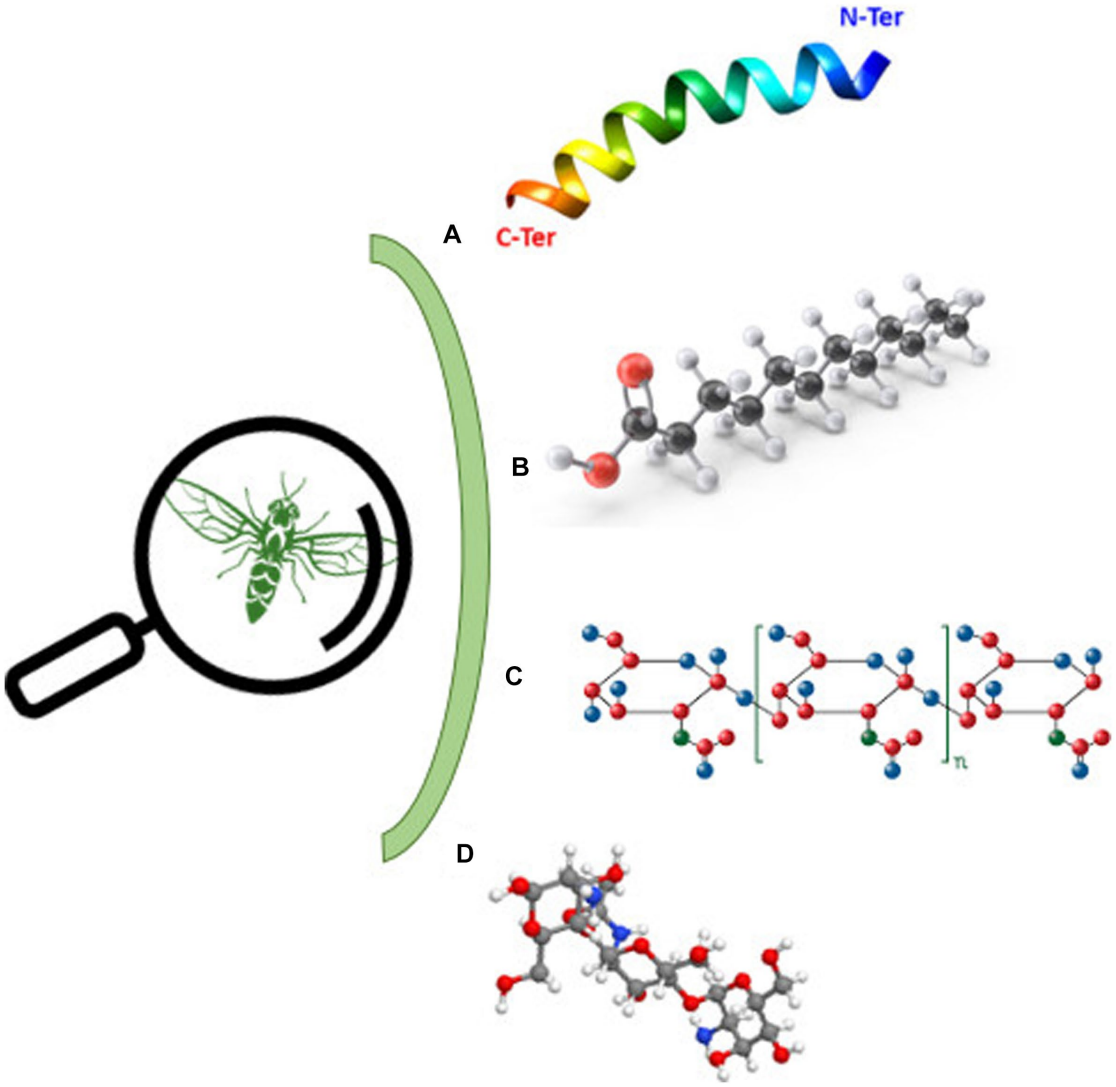
Bioactive molecules derived from insects. **(A)** Antimicrobial peptides, **(B)** lauric acid, **(C)** chitin, and **(D)** chitosan.

Bioactive substances derived from insects can be allocated into three main categories: antimicrobial peptides (AMPs), fatty acids, and polysaccharides [Figure 2 (80)].

### 4.1. Antimicrobial peptides

AMPs have received great attention as natural antibiotics that can likely confer protection against the development of bacterial resistance. Nisin is the most explored bacteriocin. In addition to its use as a preservative in several human foodstuffs, animal feed is supplemented with nisin as an anti-bacterial additive. In broilers, nisin was shown to reduce the number of Bacteroides and Enterobacteriaceae in ileal digesta (81, 82). Choi et al. (83, 84) assessed the effects of dietary administration of the antimicrobial peptide P5 (AMP-P5) and antimicrobial peptide-A3 (AMP-A3) in Ross 308 broilers. They concluded that supplementation with 60 mg AMP-P5/Kg or with 90 mg AMP-A3/Kg allowed for improved growth performances, nutrient retention, intestinal morphology, and reduced coliforms in intestines and excreta. They concluded that both of these AMPs can be used as potential antimicrobial growth promoters. In another study (85), Wen and He investigated the effect of the supplementation of synthetic cecropin (an insect-derived AMP) in broilers. The authors reported a decrease in the aerobic bacterial counts in jejunal and caecal digesta in a dose-dependent manner, as well as an improved height of the duodenal villus. Cecropin was then suggested as a possible alternative to antibiotic growth promoters in broiler production. Interestingly, the scientific literature implies that AMPs improve the intestinal health of the host animal by creating a microbial ecology, allowing it to deteriorate harmful microorganisms and favor the proliferation of beneficial ones. In this trend, Wang and his collaborators (86) described that the AMP sublancin significantly decreased the cecal count of *Clostridium perfringens* in broilers and enhanced the *Lactobacilli* count under lincomycin treatment (an antibiotic used to treat severe bacterial infections). These findings highlight the advantage of the use of insect-derived AMPs, as compared to traditional antibiotics, in the modulation of the gut microflora.

Insect-derived AMPs include α-helical peptides, cysteine-rich peptides, proline-rich (PR) peptides, and glycine-rich (GR) peptides. Most of them are cationic molecules. AMPs target the anionic phospholipids and phosphate groups of the lipopolysaccharides (LPS) of Gram-negative bacteria, and the peptidoglycan layer of Gram-positive bacteria (80). They bind to these specific sites and disturb the cell membrane of the pathogen by creating ion channels or transmembrane pores, leading to the killing of bacterial cells (87).

The α-helical peptides contain nine amino acid peptides in their α-helix region (LLCIALRKK) that are responsible for the antimicrobial activity (88). In a mouse model, acute intestinal inflammation caused by *Clostridium difficile* infection was treated by administrating an analog of the α-helical peptide. The treatment was shown to be efficient against inflammatory diarrhea associated with *Clostridium difficile* infection and *Pseudomembranous colitis* in mice (88).

Cysteine-rich peptides or insect defensins are cationic polypeptides, composed of 34 to 51 amino acids, containing multiple cysteine residues, and have three or four disulfide bridges (89). Cysteine-rich peptides are produced by fat body cells and hemolymph cells in insects. They essentially target Gram-positive bacteria (90). Some Cysteine-rich peptides were reported to be active against Gram-negative bacteria such as *E. coli* (91), as well as fungi (92), yeasts, and protozoa (93, 94). Some maggot-based medical preparations are commercially produced and used by professionals in wound disinfection and healing (95). Maggot secretions were reported for their antibacterial activity. They contain a defensin called lucifensin, which was shown to be active against *Staphylococcus aureus, Staphylococcus carnosus*, *Streptococcus pyogenes,* and *Streptococcus pneumoniae* (96).

Proline-rich peptides are cationic peptides composed of high proline residues, often associated with arginine residues in repeating motifs. PR peptides produced by insects are usually composed of 20 to 35 amino acid residues. They are mainly active against Gram-negative bacteria. They infiltrate the outer membrane and enter the periplasmic space where they impede the intracellular processes of the pathogen’s cell (97). They can also interfere with the synthesis of DNA and RNA by binding to nucleic acids (98, 99). PR peptides were described to exhibit antimicrobial effects in *E. coli*, *Klebsiella pneumoniae*, *P. aeruginosa*, and *Acinetobacter baumannii* (100, 101). Apidaecins are a group of PR peptides that destroys many Gram-negative bacteria, and hence, may be used as new candidates for peptide antibiotics (98).

Glycine-rich peptides include many molecules such as sarcotoxin IIA, hymenoptaecin, attacin, diptericin, and coleoptericin. They are rich in glycine residues (14 to 22%) and are active against fungi, Gram-negative bacteria (90), and cancer cells (102). The high content of glycine plays a key role in the tertiary structure of GR peptides and their mechanism of action against pathogens (103).

### 4.2. Fatty acids

Lauric acid (LA, C12:0) is a saturated medium-chain fatty acid (MCFA) with a 12-carbon backbone. MCFAs were reported for their antimicrobial activity and suggested for preventing and treating gastrointestinal disease in piglets after weaning (104), as well as pig enteritis (105). MCFAs are also used directly by enterocytes to produce energy, thus they help to strengthen gut integrity in young piglets (106).

In Cobb 500 broiler chickens, supplementing 22.8 g of free LA per 100 g of total fatty acids allowed enhancing feed: gain ratio and breast meat yield (107). In male Ross 308 broiler chickens, LA significantly ameliorated BW and ADG (108). The intestinal mucosal barrier was enhanced and the immunoglobulins (IgA, IgM, and IgY) were upregulated. Regarding inflammatory cytokines, IL-1β, IL-6, TNF-α, IL-4, and IL-10 were downregulated (108). Concerning lipid metabolism, the levels of phosphatidylcholines were decreased, those of lysophosphatidylcholines were increased and the sphingolipid metabolism pathway was inhibited (108). Moreover, *Phascolarctobacterium*, the *Christensenellaceae_*R7 group, and Bacteroides were reduced, and *Faecalibacterium* and *Ruminococcaceae* UCG-014 were increased, indicating the ability of LA to significantly (*p* < 0.05) regulate cecal microbiota composition (108). These findings argue for the potential of LA as an additive in poultry feed and suggest a new alternative for antibiotics in poultry husbandry and a new way to ensure feed safety.

Lauric acid has been described to have antiviral and antibacterial activities (109). It is active mainly against Gram-positive bacteria, even in the presence of solid particles and at a pH greater than 6 (110). In animal models, C12:0 was effective in pigs with D-streptococcal infections (111). It is important to add that monolaurin, a monoester from lauric acid, can be formed, and has a biological bactericide activity greater than lauric acid (109).

Undissociated forms of MCFA can infiltrate the lipid membrane of bacteria, and move to the cytoplasm where they dissociate and decrease the pH. In an attempt to maintain its neutral pH, the bacterial cell exports many protons. Cellular ATP is then consumed, leading to energy depletion and bacterial cell death (112, 113).

Lauric acid was detected in high levels (up to 60%) in BSF prepupae reared in organic waste streams with high amounts of starch (114), but it is important to note that this level depends on the rearing substrate and the insect feed (115). In order to gain maximum benefit from the antimicrobial effects of lauric acid, it is recommended to feed whole larvae or prepupae to poultry. The utilization of less sustainable protein and fat sources could therefore be decreased.

### 4.3. Polysaccharides

Both chitin and chitosan are polysaccharides available in high amounts in insects. Chitosan, β-(1–4)-linked 2-amino-2-deoxy-β-D-glucopyranose, is an N-deacetylated derivative of chitin, and the main organic skeleton material of the exoskeleton of insects (116). In chitin, the acetamide groups are converted into primary amino groups (117).

Chitosan was reported to act as a chelating agent in biological systems and exhibited antimicrobial activity against bacteria, yeasts, and fungi (118). Indeed, it is considered a potential target for recognition by the immune system in many species, including birds (119). In other words, the innate immune system is stimulated by chitin and chitosan from insects (120, 121). In this context, a decreased albumin/globulin ratio was recorded in broiler chickens fed MW larvae, indicating an improved immune status (11, 122). Furthermore, improved immune activity was seen in laying hens fed BSF larvae meal-based diet with 1 g of chitin offered daily (123). In their trial, Islam and Yang (121) described decreased caecal *E. coli* and *S. enteritidis* contents in broilers fed 0.4% dry MW or Z. morio larvae, associated with an increase in IgG and IgA levels, which play a protective role against microbial infections. Interestingly, chitin is known for its hypolipidemic and hypocholesterolemic properties (124). In broiler chickens, chitin supplementation resulted in a decrease in body fat and possibly the production of leaner meat (125). Moreover, chitin and chitosan were recognized for their antiparasitic activity against *L. major* in mice (126) and intestinal infections with *Eimeria papillata* (127).

Chitin and chitosan exhibit their antimicrobial and antiparasitic effects directly or indirectly by supporting immune activity. The direct mechanism resembles that of AMPs and induces cell lysis, penetration of cytoplasmic membranes, and cation chelation (128). It is worth stating that chitosan was shown to be more effective in treating microbial infections, while chitin was more effective in treating parasitic infections (129), but they have in common their immunomodulatory effects. Immune cells can recognize both of these polymers thanks to their galactin-3, mannose receptor, RegIIIγ, dectin-1, and various toll-like receptors. These receptors have been shown to interact with chitin and/or chitosan (119).

Health benefits attributed to insects can create an additional value chain for poultry health and be antibiotic alternatives. The antimicrobial properties and the immuno-modulating effects of insect-derived bioactive molecules may contribute to enhancing global health, reducing the use of antibiotics, and avoiding antibiotic resistance when these insect products are included in poultry diets.

## 5. Growth performances

Insects are a sustainable, attractive, safe, and promising alternative protein source that could satisfy the world’s growing demand for food. This is especially true for poultry nutrition. Housefly larvae and pupae have been widely investigated in broilers (130–134). The different studies agreed that this protein source, either fresh or dried, can be used as an alternative to fish meal, soybean meal, and other protein ingredients. No adverse effects were registered regarding survival, daily FI, BWG, and FCR. It is worth stating that combining housefly meals and conventional meals in the same diet allowed for enhanced growth performances (130, 134). This may point toward a more balanced feed when comparing conventional and insect meals. Furthermore, housefly meal can partially or totally replace FM in broiler diets. Inaoka et al. (135) and Awoniyi et al. (130) reported that housefly maggots and pupae may be included in the diet up to 7%. If the inclusion rate exceeds 10%, a decrease in FI and growth performance was observed. This seems to be related to the dark color of the produced feed, which is not attractive to chickens (136, 137), and to the imbalanced AAs profile (5). In this latter case, methionine supplementation might improve broiler growth performance. Nevertheless, Hwangbo et al. (64) concluded that growth performance was enhanced when supplementing broiler feed with 10–15% housefly larvae. Similarly, Okah and Onwujiariri (133) described that the experimental group of chickens fed 20 and 30% of housefly maggot meal registered the best BW, as compared with the control group. It seems that this discordance regarding the inclusion rate is related to the processing method of the insects, which influences the availability of nutrients, as well as the inclusion rate of the insect meal.

Mealworm meal is also an alternative protein source that can be included in poultry feed without affecting the growth performance of the birds. In broilers, and without affecting feed intake, increasing levels of MW meal (from 0.1 to 0.3%) allowed significant improvement (*p* < 0.05) in both the BWG and the FCR from 1322.0 g to 1423.3 g, and from 1.88 to 1.75, respectively. The highest dressing percentage was recorded in the group supplemented with 0.3% MW (39). Interestingly, and according to this latter study, the gross return and the net profit were significantly (*p* < 0.05) higher for the group fed 0.3% MW (0.41 US$). The control group registered the lowest return (0.34 US$). The findings of this study were corroborated by Ramos-Elorduy et al. (53) who concluded that MW, once incorporated in the broiler diets up to 10%, has no negative effects on BWG, FI, and FCR. These authors noted a consequent decrease in the inclusion rate of SBM from 31 to 20% after incorporating dried yellow MW up to 10%, without any significant differences in the performance results. According to Schiavone et al. (138), the best BWG was obtained using 25% MW. Another assay aiming to fully substitute SBM by MW larvae in the broilers’ diet was conducted by Bovera and his collaborators (6). No significant effects on growth performance, carcass traits, and meat quality were observed. In addition, the FCR was significantly enhanced (*p* < 0.05) in the MW group. As compared to the control group (SBM group), broilers fed MW as a unique protein source had a more developed digestive system, a greater percentage of the spleen, longer intestines, ileum, and ceca (p < 0.05). Compared with an iso-proteic and isoenergetic SBM diet, MW larvae did not affect the FI of broilers. Indeed, a better FCR was calculated in the experimental group (11). Importantly, the lowest albumin/globulin ratio was recorded in broilers that received MW diets, which indicates a better disease resistance and immune response in birds. This finding may be attributed to the prebiotic effects of chitin and other bioactive molecules produced by the insect. Furthermore, the values of aspartate aminotransferase of the MW group were within the normal range for broilers and were not accompanied by an increase in gamma-glutamyl transferase, lactic dehydrogenase, or lactic dehydrogenase. Such results indicate that no alteration occurred in liver and muscle cells, and suggest that MW larvae can be used as a unique protein source in broiler diets.

BSF is another potent candidate to be included in poultry feed as SBM and FM substitute. The nutritional profile of these worms is comparable to that of FM and, in some aspects, better than that of SBM. Maggot meal could replace FM, without altering BWG, FI, and FCR. As a substitute for SBM, dried BSF larvae allowed for decreasing FI and enhancing FCR, without adverse effects on BWG (5). In growing broiler quails, no significant statistical differences were reported regarding BWG, FI, FCR, and mortality rate between control, 10% defatted BSF larvae meal, and 15% defatted BSF larvae meal groups (65). Regarding palatability, no special preference for broilers was shown toward control or defatted BSF larvae meals. It is interesting to mention that in this latter study, 10% defatted BSF larvae substituted for 28.4% of soybean oil and 16.1% of SBM, and 15% of defatted BSF larvae meal allowed the replacement of 100% of soybean oil and 24.8% of SBM. It can be concluded that BSF meal is a good alternative to FM and SBM in broiler diets, without any negative impacts on the growth performances of the birds.

Grasshoppers were also assessed in broilers’ feed. Grasshopper meal was used to replace 50 and 100% of FM (the inclusion rate was 5 and 10%, respectively) in Arbor Acres broiler chickens, which exhibited improved growth (29). These findings were corroborated by the findings of Sanusi et al. (139) and Sun et al. (140) who reported that grasshopper meal could totally replace FM without any adverse effects in Anak 2000 broiler chickens. However, diets containing more than 25% wild edible grasshopper (*Ruspolia nitidual*) meal were found to lower FI in indigenous chickens (141).

Earthworms were also recommended as a potential protein source that could replace FM and SBM in poultry feeding. Loh et al. (142) reported that incorporating EWM in Ross male broiler chickens diets at 5, 10, 15 and 20% allowed enhancing FI (*p* < 0.05), increasing BW (p < 0.05), improving FCR (p < 0.05) and reducing fecal lactic acid bacteria count. Other related studies confirmed that Ross 308 broiler chickens fed on diets containing 2, 3%, or 4% EWM increased their BW and FI (143–145). An inclusion rate of 5% EWM (*Eudrilus eugeniae*) enhanced FCR (87). Lower inclusion rates (1% EWM and 1% *vermi-humus*) resulted in a negative impact on BWG, despite the observed improvements in the immune functions in broilers (145). This was probably due to the unbalanced amino acid profile of the diet.

## 6. Carcass traits

The effect of insect meal feed on the carcass quality of broilers is quite different. Although some studies did not disclose any significant impact, several authors reported enhancing effects. Hwangbo et al. (64) did not find significant differences in carcass traits of Ross broiler chickens when replacing SBM with HF larvae or pupae. The trial used 600 one-day-old male chicks and lasted 35 days. Similarly, Awonyi et al. (130) assessed the effect of substituting FM with maggot meal on 102 ANAK 3000-strain broiler chicks. The authors stated that the live, dressed, and eviscerated weights, as well as the relative length, breadth, and weights of the pectoral and gastrocnemius muscles, were not significantly affected by HF maggot meal diets. The dressing percentage was not affected as well. Nevertheless, Hwangbo et al. (64) described higher dressing percentages in broilers fed 5 to 20% HF meal. Likewise, Pieterse et al. (146) reported that unsexed Ross 308 broiler chickens fed HF meal had heavier carcasses and higher breast and thigh muscle weights than those fed SBM-based diet.

Several studies (147–149) have been conducted in order to investigate the effect of using MW on growth performance and carcass traits of male Shaver brown broiler chickens, female Label Hubbard hybrid chickens, and male Ross 708 broiler chickens. The authors concluded that using MW meal in broiler diets had no significant effects on carcass traits. However, Ballitoc and Sun (150) found that unsexed Ross broiler chickens fed on a diet supplemented with 2% MW meal over 35 days exhibited an increased carcass yield, slaughter weight, dressed weight, eviscerated weight, and a reduced abdominal fat weight. In the same way, Hussain et al. (39) reported that broiler chickens fed a diet containing 3% MW meal exhibited increased dressing percentage, feed cost, gross return, and net profit.

With regard to BSF meal, Cullere et al. (65) have found that both breast meat weight and yield did not differ among control and experimental groups in growing broiler quails when replacing FM with 10 and 15% BSF meal in broiler quails diets (a total of 450 10-day-old *Coturnix coturnix japonica* chicks were used in this study). Another similar finding was reported by Schiavone et al. (61) who showed that a diet containing 100% BSF fat as a substitute for soyabean oil did not affect the carcass quality of Ross 308 broiler chickens. Nonetheless, Altmann et al. (151) reported that Ross 308 male birds fed up to 15% BSF meal over 35 days had heavier carcasses compared to those fed an SBM-based diet. In another trial conducted on 256 one-day-old male broiler chicks for 35 days, it was reported that the diet containing 5% BSF meal resulted in a reduction of the abdominal fat percentage, and 10% BSF meal resulted in heavier carcass weight and greater breast percentage (152). Carcass composition was also improved in Hubbard S757 broilers fed 7.8% BSF meal in combination with 5.2% alfalfa meal (153).

Regarding silkworms, Khatun et al. (154) reported that increasing the levels of SW meal in replacement of SBM in poultry diet allowed increasing linearly the FCR, dressing percentage, and profitability in broilers. Ullah et al. (155) described that the FCR and the dressing percentage were not affected by increasing levels of SW (from 25 to 100%) in broilers. Hence, it can be concluded that SWM can be used to substitute FM or SBM without adverse effects on broiler performances, carcass traits and profitability. The literature explained that the improved broiler production traits could be related to the higher content of EAA in SWs, and to the growth prompting factors they contain (156), such as the ecdysteroid activity (a hormone involved in protein synthesis and tissue formation). However, this latter argument needs to be elucidated.

Live grasshoppers were also reported to improve live weight and carcass composition, and total lipids phospholipids, and anti-oxidative potential of the meat, of Qinjiaoma broiler chickens (85). In two other studies (56, 157), the effect of replacing FM with grasshopper meal (50 and 100%) on the carcass traits and the profitability of broiler chickens was assessed. The carcass analysis showed significant differences (*p* < 0.05) between the control and experimental groups, with the exception of breast, pancreas, proventriculus, heart, spleen, liver, lungs, and crop and chest weights. The highest profitability was calculated for the group fed 100% grasshopper meal, followed by the group that received 50% of this insect meal, followed by the control group. These literature reports imply that grasshoppers can substitute for FM without negative effects on carcass traits and economic return in broiler chickens. These findings may be attributed to the enhanced CP and EE values of the insect-based diets and their improved digestibility as well.

## 7. Meat quality

A general improvement in meat quality in broiler chicken was observed when feeding insect meal-based diets. Hwangbo et al. (64) described enhanced contents of lysine and tryptophan in the breast muscle, despite the unchanged content of CP. This may be due to the greater AA profile and the high protein digestibility (98.5%) of HF larvae meal. In the same context, Sun et al. (140) reported that a live grasshoppers-based diet improved the total lipids, phospholipids, and antioxidant potential of broiler chicken meat. In addition, a full replacement of FM by EW meal resulted in a reduced fat content in the breast and thigh meat of broiler chicken (43). The antioxidant capacity of the meat was improved using a diet containing 5% EW meal. An inclusion rate of 7% EW meal allowed for obtaining greater aroma, juiciness, residues, and flavor in Cobb 500 broiler meat (144). Similarly, Gholami et al. (143) concluded that feeding EW meal to Ross 308 broiler chickens increased breast meat yield, but had no effect on thigh and abdominal fat percent.

Cullere et al. (65) reported that BSF meal feed did not affect the breast meat weight of broiler quails, and the meat quality remained unchanged (61). In the same way, Leiber et al. (153) described an improved meat redness using BSF meal in Hubbard S757 broilers. Schiavone et al. (152) noted that feeding 5% BSF meal lowered the abdominal fat percentage, 10% BSF meal resulted in greater carcass weight and breast percentage, and 15% BSF meal resulted in an increased abdominal fat percentage, meat redness, meat protein percentage, MUFAs percentage in breast meat, and reduced breast meat PUFAs percentage. The diet containing 100% BSF fat generated an elevated content of saturated fatty acids (SFAs), and a reduced level of PUFAs in the breast meat. In fact, chickens assimilate unsaturated fatty acids (UFAs) better than SFA. UFAs form mixed micelles with monoglycerides and conjugated bile salts. They are transported to the surface of the intestinal mucosa where they are absorbed (158). PUFAs play a myriad of roles in the body and are precursors of cellular functional molecules (159). It was shown that BSF oil supplementation increased n-3 and n-6 fatty acids in the breast meat of chickens (62), without influencing the level of MUFAs (61).

Several studies agreed that insect meal feed did not have any negative effect on poultry products Hussain et al. (39) indicated that MWs, when used in broiler chicken feeding, do not affect the organoleptic properties of the meat (i.e., taste, tenderness, juiciness, flavor, and color). Similarly, SW meal does not impact the taste of poultry products (13) and does not cause a fishy taint in the meat (160). Furthermore, HF meal fed to Cobb 500 broiler chickens enhanced meat flavor, meat juiciness, meat aroma, meat tenderness, and meat desirability (27). The breast muscle yield and the water-holding capacity were improved as well, and the thawing loss and cooking loss were decreased (146). Regarding color, it is known that the pigments contained in the feed originate from the raw materials and ingredients used in feed formulation.

The pH value is an important parameter for the detection of meat defects such as pale, soft, and exudative meat (PSE). This anomaly occurs when the post-mortem pH value measured 15 min after slaughtering is lower than 5.6. Cullere et al. (65) reported a pH value of 5.67 using an HF meal-based diet, and Bovera et al. (147) measured higher pH values in the poultry group fed insect meal-based feed. Pieterse et al. (161) concluded that there are no differences regarding the initial and ultimate pH of the thigh muscles between broiler chickens fed a control diet and those fed a BSF meal-based diet. It can be concluded that feeding insect meal-based diets to broiler chickens does not cause abnormal pH values, and consequently are not responsible for causing meat defects.

It seems that insect meals used in broiler chickens have the potential to produce meat with comparable chemical and quality traits compared to those fed diets containing traditional feed ingredients.

## 8. Laying hens and egg quality

In addition to broilers, BSF was also investigated in laying hens as a substitute for SBM and FM. The inclusion rates varied from 5 to 100% (10, 13, 162, 163). In the assay carried out by Maurer et al. (164), SBM was partially (50%) and completely (100%) replaced by dried and defatted BSF larvae meal in a laying hens diet. No significant differences were noted between the control and the experimental groups with regard to egg production, FI, egg weight, and feed efficiency. Although there was a trend (*p* = 0.06) for lower albumen weight in the 24% BSF group, there were no differences in yolk and shell weights. Kawasaki and his collaborators (165) investigated the effect of BSF larvae and pupae meals on the egg quality of laying hens. They reported that the highest weights of egg and albumen were recorded in the poultry group fed BSF pupae. The eggshell thickness remained unchanged between these two experimental groups. Compared to the control group, feeding BSF meal improved the eggshell thickness, probably due to its high content of available calcium (163, 166). In another study, laying hens receiving a diet containing 10% BSFM have shown an increased egg weight, albumen weight, eggshell thickness, albumen height, and plasma calcium. The egg yolk color score was enhanced as well (165). An inclusion rate of 3% BSFM in Xuefeng black-bone laying hens improved egg weight; Haugh unit; eggshell weight; yolk C14:00, C17:00, and C20:2 fatty acids; yolk amino acids (glutamic acid, methionine, phenylalanine, and leucine), plasma total superoxide dismutase; and plasma avian influenza virus antibody. However, eggshell thickness and plasma interleukin-2 decreased (23). It is also important to note that feeding Lohman Brown Classic laying hens a diet with 17% BSFM to substitute for SBM resulted in poor growth and production percentage, as well as a reduction in blood lipids, blood chloride, and blood creatinine. However, an increase in the percentage of small, medium, and extra-large size eggs, blood globulin, blood calcium (123), and fecal dry matter (164) was noted.

HF maggots and pupae were assessed in rearing layers (167, 168). In Isa brown and Nera black layer hens, HF larvae meal was used as a 50% (7.08% HF meal and 1.50% FM) and total (9.44% HF meal and 0% FM) replacement of FM. This study showed that the experimental diets did not affect feed intake (FI) and feed conversion ratio (FCR) but significantly (*p* < 0.05) improved hen-day production, from 3.00% for the FM group to 4.72% for the insect meal group (168). This result seems to be attributed to the enhanced AA profile of the diet when HF larvae meal and FM were supplied together. No differences between the control and the experimental diets were reported by the authors regarding the egg quality traits. Nevertheless, the shell thickness weight was decreased in the experimental groups (168). This reduction may be explained by the lower calcium content of HF larvae meals. Moreover, Dankwa et al. (167) found that supplementation with 30–50 g of maggots results in a significant (*p* < 0.05) improvement in clutch size, number of hatched eggs, and egg and chick weight. The results of the above studies suggest that HF meal could be used as a partial replacement for FM in laying hens’ feed.

Grasshoppers (*Ornithacris cavroisi*), silkworms, bee propolis, and pollen were also investigated in laying hens’ diets. An enhanced Haugh unit in Isa Brown laying hens was observed when feeding 25% grasshopper meal as a substitution for FM. The egg yolk color was improved using an incorporation rate of 75% grasshopper meal (29). White Leghorn layer hens fed 5.6% SW meal as a replacement for SBM showed a significant (*p* < 0.05) improvement in live weight, FCR, and egg production, with a significant (*p* < 0.05) reduction in FI and feed cost (169). An inclusion rate of 8% SWM was reported to improve survivability (169). Bee propolis was included at 0.025 and 0.05% in Lohmann LSL laying hens’ diets. The results showed an increase in egg mass and production, Haugh unit, albumen height, yolk height, index and weight, blood total protein, globulin, hemoglobin, and lymphocytes. A simultaneous decrease of FCR, yolk diameter, blood cholesterol, heterophil, and lymphocyte ratio was reported (170). Bee pollen was supplemented to Sinai laying hens at 0.05 and 0.15%. A subsequent increase in the egg number and mass, production percentage, feed intake, red blood cells, white blood cells, and lymphocytes was described. But BW, BWG, heterophils, heterophil lymphocyte ratio, blood cholesterol, and blood triglycerides were reduced (171).

## 9. Insect farming and processing: interactions with the nutritional value

Food security arguments for switching to an alternative protein source are powerful motivators for using insects in poultry diets, especially since scientific reports have approved insect meals as a valuable and sustainable protein source that is able to meet the production requirements of these birds. However, the nutritive value of insects varies throughout the production chain, from the insect-rearing step to the feed-manufacturing phase.

Insect farming conditions have critical effects on the nutritional value of the insect larvae. In fact, larval density as well as the quantity and the quality of the insect diet determine the body composition of the larvae. Barragan-Fonseca et al. (172) investigated the effect of dietary nutrient concentration and larval rearing density on the growth performance and the chemical composition of BSF larvae. Four (04) rearing densities (50, 100, 200, or 400 larvae per container) and three nutrient concentrations (low, medium, and high) were tested. The authors reported that individual larval weight and total larval yield increased in all groups that received high nutrient concentration, in spite of the larval density. Larval crude fat content was higher in groups with low rearing density and received high nutrient concentrations. Larval crude protein was higher in groups at low rearing density and low nutrient concentration. This study revealed that larval CP content is affected by nutrient concentration, rearing density, as well as their interaction. Indeed, this study suggested that larval CP content is regulated within narrow limits, and larval crude fat depends on the nutrient concentration and the larval density. In fact, it was stated that high-fat and high-carbohydrate diets increase larval crude fat content (173) compared to low-fat and/or high-fiber meals which decrease the larval crude fat content (174, 175). These findings highlight the importance of rearing practices and insect diets in producing larvae with valuable nutritive profiles.

In animal feed, insects are usually added as a meal. Insects are first dehydrated or roasted and then ground into a fine powder, the meal (176). Some popular processing methods include steaming, boiling, frying, smoking, drying, and toasting (177). Although a significant reduction of microbial hazards can be ensured through thermal treatments, the nutritive value of the insects can be affected. Nyangena et al. (178) demonstrated that all thermal treatments (boiling, toasting, oven-drying, and their combinations) except solar-drying lowered bacterial counts and eliminated yeast and molds in BSF prepupae. Meanwhile, these heat treatments decreased the crude fat content up to 14.3–28.2 g/Kg DM in the order: toasting > boiling > oven-drying > solar-drying. They also increased the CP content up to 37–41.3 g/Kg DM in the same order and improved the available carbohydrates. Another study conducted by Dobermann et al. (179) examined the effect of freeze-drying and heat processing at low and high temperatures (45°C and 120°C) on the nutritional profile of the black cricket *Gryllus bimaculatus*. The results showed that drying at 45°C obtained high protein and calcium contents. Moreover, freeze-drying conserved long-chain polyunsaturated fatty acids, more than drying at 120°C. Consequently, more attention is needed in order to protect the nutritive value of insects during heat processing.

As in the case of soy and whey protein concentrates and isolates, developing insect protein powders involves defatting, protein solubilization and recovery, purification, and drying (180). The methods used for chitin separation include enzymatic proteolysis, thermal treatments, solvent extractions, or alkali/acidic reactions. Free amino acids and peptides of different sizes are then produced. These processes may modify the insect proteins and affect their functionalities through structural modifications, molecular weight decreases, or polarity increases (181). Longer proteolysis and a high enzyme concentration result in lower molecular weight peptides with affected techno-functional properties. Also, the selection of the enzyme protease will determine the peptide amino acid sequence and/or amino acid residues which are responsible for the nutritional properties of the protein hydrolysates (182).

Fat extraction is a critical step to produce insect proteins and to obtain a high yield of good quality insect fat. Conventional extraction methods include chemical extraction using ethanol and methanol as extraction solvents, mechanical oil press, and three-phase partitioning (TPP). Non-conventional extraction techniques involve supercritical CO_2_. Laroche et al. (183) compared the effects of conventional solvents, TPP, and supercritical CO_2_ on the fat extraction yield and the fatty acid profiles of cricket and mealworm meals. They reported that supercritical CO_2_ was efficient only in cricket meal, whereas ethanol extraction and TPP increased the fat yield from both meals. Regardless, the defatting method should be selected with care.

## 10. Safety issues of insects as feed

Producing safe insects as an alternative protein source in poultry feeding is a challenge. The safety of edible insects always needs to be guaranteed. Contamination is very likely to occur and to be transferred to the animals unless safety rules are not ensured during the industrial rearing cycle. Insects for food have been associated with *Staphylococcus aureus*, pathogenic *Clostridium* spp., and pathogenic species of the *Bacillus cereus* group. When regarding insects for feed, microbes from the substrate can enter the gut, proliferate and become part of the gut microbiota (184, 185). The bacterial species vary depending on the region of the gut they colonize since each region has its distinct properties, and represents, therefore, a different ecological niche (184). Regarding the BSF, a set of insect species were screened, including *Actinomyces* sp., *Dysgonomonas* sp., *Enterococcus* sp., and *Morganella* sp. It is important to note that these species have been tracked in other insects as well (186). Regarding industrially produced HF, there are no studies that have been performed to investigate its microbiota to our current knowledge. Little is known about other types of biological contaminants, fungi, viruses, protozoa, and prions.

Substrates and feed for insects can be adulterated with heavy metals, pesticides, fungi, and bacteria. Some studies reported potential risks in BSF larvae. Wynants et al. (187) observed contamination with *Salmonella enterica serovar* Agona in the rearing residue. Wu et al. (188) and Jiang et al. (185) reported an infestation by *Campylobacter* and *Clostridium* species, respectively. Presumptive *B. cereus* was revealed in the BSF larvae with counts up to 6,000 cfu/g. With respect to viruses, Chen et al. (189) isolated an Escherichia phage from the BSF larvae gut. Indeed, prions can infest non-processed insects from contaminated substrates, even insects were shown to be not able to produce them (190).

Mycotoxins from feed or rearing substrates are another potential risk that can affect growth and increase the mortality of insects. Consuming a mycotoxin-contaminated insect meal can affect poultry health and performance. Schrogel and Wätjen (191) found that the mycotoxin content of the substrate was 25-fold higher than the maximum limit. Therefore, they recommended starving insects for 24 h before harvesting. In addition, insects may accumulate not only residues of pesticides, veterinary drugs, and hormones but also dioxins and PCBs as well (192).

Another potential risk is the contamination of feed and/or substrate with heavy metals (e.g., cadmium, arsenic, cobalt, copper, nickel, etc.). In fact, Vijver et al. (193) proved the accumulation in BSF larvae of cadmium, copper, lead, and zinc from the soil. Schrogel and Wätjen (191) stated that HF is able to concentrate cadmium, whereas BSF is able to accumulate arsenic. Insect larvae are able to accumulate cadmium or plumbum in their exoskeleton (194).

The presence of pathogenic bacteria in insects can be avoided through farming or processing conditions (195). Rearing insects on pollutant-free substrate is key for preventing insect and animal contamination.

## 11. Conclusion and perspectives

Insects can fulfill a major role in upgrading the value of poultry feed, coping with competing uses of food system resources, and contributing to food safety. Insect meal represents an alternative protein source enriched with highly digestible essential amino acids, fat, and other important nutrients such as MUFA, PUFA, calcium, phosphorous, copper, iron, magnesium, manganese, selenium, and zinc, in addition to riboflavin, pantothenic acid, biotin, and, in some cases, folic acid. However, the quality of insect meals and their nutrient profile depends on the rearing medium, insect feed, and processing methods. Insect farming is able to provide an alternative source of income for small-scale farmers and large-scale industries. Nevertheless, the cost of production needs to be reduced. Also, the risk of contamination and the spreading of pathogens needs to be kept under control. Beyond their nutritional value, insects produce bioactive compounds that could act as health stimulators in livestock. Hence, insects may be used as antibiotic alternatives because of the biological properties of their AMPs and their synergic effects with fatty acids and either chitin or chitosan. They may be also used in the future to combat antibiotic resistance. With the unceasing reliable advancements in scientific research in this field, insect protein is being touted as the answer to a consistent and tenable feed group, and more importantly, it is predicted to be the number-one sustainable protein source for the poultry feed industry in future generations.

## Author contributions

IBS and HY: conceptualization. IBS, TN, and NM’H: validation. IBS, HY, and MBL: resources and writing–original draft preparation. IBS and NM’H: writing–review and editing. TN and NM: supervision. All authors contributed to the article and approved the submitted version.

## Conflict of interest

The authors declare that the research was conducted in the absence of any commercial or financial relationships that could be construed as a potential conflict of interest.

## Publisher’s note

All claims expressed in this article are solely those of the authors and do not necessarily represent those of their affiliated organizations, or those of the publisher, the editors and the reviewers. Any product that may be evaluated in this article, or claim that may be made by its manufacturer, is not guaranteed or endorsed by the publisher.
